# Interventions to address antimicrobial resistance in migrants: a systematic review

**DOI:** 10.1093/jacamr/dlag059

**Published:** 2026-04-30

**Authors:** Lucy Lammie, Amelia Williams-Walker, Laura B Nellums, Catrin E Moore

**Affiliations:** Institute of Infection and Immunity, St George’s School of Health and Medical Sciences, City St George’s University, London, UK; Institute of Infection and Immunity, St George’s School of Health and Medical Sciences, City St George’s University, London, UK; College of Population Health, University of New Mexico, Albuquerque, NM, USA; Institute of Infection and Immunity, St George’s School of Health and Medical Sciences, City St George’s University, London, UK

## Abstract

**Background:**

Antimicrobial resistance (AMR) is a major global health threat that disproportionately affects structurally marginalized groups, including migrants. Yet migrants are largely absent from AMR surveillance systems and response strategies, creating health inequities and undermining global containment efforts. This systematic review examines the evidence base for interventions targeting AMR in migrant populations, with three objectives to summarize existing research; to identify gaps in data collection, intervention design and outcome measurement; and to propose a path towards more equitable, migrant-inclusive AMR responses.

**Methods:**

We systematically searched MEDLINE, Embase and PubMed (January 2000−December 2025), following PRISMA guidelines using terms related to migration, AMR and interventions. Tuberculosis studies were excluded and we prioritized interventions addressing WHO Critical and High-Priority pathogens.

**Results:**

Of 4039 records screened, only four studies met inclusion criteria, therefore we performed a narrative synthesis.

**Discussion:**

Across these studies we identified three persistent gaps: (i) surveillance and monitoring rarely stratified data by migration status, ethnicity or country of origin; (ii) interventions were almost exclusively hospital-based, with little attention to community settings or care pathways; and (iii) outcome measurement was limited, with a narrow focus on microbiological endpoints and few robust clinical or equity-sensitive outcomes. Current evidence on AMR interventions for migrants is scarce, fragmented and insufficient to guide policy. Future work should prioritize high-quality, disaggregated surveillance data, contextually appropriate study designs and community-based care models, enabling rigorous, equity-driven evaluation of interventions.

## Introduction

Antimicrobial resistance (AMR) is a growing global health crisis, recognized by the World Health Organization (WHO) as one of the top 10 worldwide health threats.^1,2^ As resistance rises, it risks significant impact on morbidity, mortality and healthcare costs.^3,4^ If left unaddressed, the economic and social consequences are severe, with the World Bank warning that AMR could push 28 million people into extreme poverty by 2050.^5^

The burden of AMR is not evenly distributed. Evidence increasingly shows that structurally marginalized populations bear a disproportionate risk, including migrants, defined here as individuals living outside their country of birth.^6–8^ Migration occurs for diverse reasons and under varied legal statuses, but common barriers, including legal exclusion, language barriers, precarious housing and restricted healthcare entitlements, limit access to prevention and treatment and can increase risk of transmission.^9^ Yet, migrants remain largely invisible within AMR surveillance, prevention and response strategies. This exclusion not only perpetuates inequities, but also undermines global containment efforts given the cross-border spread of resistant pathogens.^10^

While studies have highlighted the risk of AMR in migrants, it is necessary to evaluate the effectiveness of targeted interventions. This review examines strategies aimed at mitigating AMR in migrants, moving beyond descriptive epidemiology to evidence that informs clinical practice and policy. We conducted a global systematic review with a narrative synthesis of interventions, assessing their impact on measurable health outcomes. By consolidating the available evidence, we identify gaps in intervention design and propose a framework for more equitable and effective AMR responses.

## Methods

### Search strategy and selection criteria

This systematic review was conducted in accordance with PRISMA guidelines. On 7 December 2025 we searched three electronic databases (MEDLINE, Embase and PubMed) for peer-reviewed studies published between 1 January 2000 and 7 December 2025. No language restrictions were applied to ensure global scope. The search strategy combined terms relating to migration, AMR and interventions. A detailed summary of search terms and eligibility criteria are provided in the Supplementary Data (Tables S1 and S2, available as Supplementary data at *JAC-AMR* Online).

The United Nations definition of migrants being individuals living outside their country of birth, irrespective of legal status or reason for migration was adopted for this review.^6–8^ Both short-term (3–12 months) and long-term (>12 months) international migrants were eligible for inclusion. Internal migrants were excluded.

This definition was operationalized during screening by including studies in which:

1. Participants were explicitly described as migrants, refugees, asylum seekers, foreign-born individuals or internationally displaced persons; or
2. Migrant status was clearly identifiable through reported country of birth or migration status.

Studies including mixed populations (migrants and non-migrants) were eligible only if outcomes were reported separately for migrants (i.e. disaggregated data). Studies in which migrant participants were included but results were not disaggregated were excluded as the impact of interventions on migrants could not be determined.

Interventions were defined using the Effective Practice and Organisation of Care taxonomy, with a focus on WHO-designated Critical and High-Priority pathogens for AMR.^11^ For the purpose of this review, an intervention was defined as any active measure, programme or policy designed to change or influence the prevention, diagnosis, prescribing or treatment of AMR, thereby excluding purely descriptive studies focused solely on surveillance or data collection.^12^ Tuberculosis was excluded due to its distinct surveillance and treatment protocols.

### Data extraction and quality assessment

After removal of duplicates, the titles and abstracts of 4039 records were screened independently by two researchers (L.L., A.W.-W.) for inclusion. Of these, 24 articles were selected for full-text review. Screening and data extraction were performed by two reviewers, with any discrepancies resolved through consensus, and a third reviewer (C.E.M.) consulted for any disagreement. Key variables extracted included study setting, population characteristics, target pathogen(s) targeted, intervention type and measurable outcomes (Table 1).

**Table 1.** Summary of included studies addressing AMR interventions in migrants

Study (author, year)	Setting and country	Population characteristics	Pathogen(s) targeted	Intervention description	Measurable outcomes reported	Recommendations
Georgakopoulou *et al.* (2016)^13^	Transit Centre, Greece	Refugees (*n* = 16)	Shigella	Delivery arrangement: Case-finding/Syndromic surveillance and environmental hygiene measures	Limited person-to-person transmission; surveillance found feasible	Implement combined phenotypic and molecular typing (e.g. serotyping, AST, PFGE) to distinguish sporadic from outbreak cases. Use antimicrobial susceptibility testing to guide appropriate empirical and targeted therapy. Strengthen tracking systems to monitor treatment outcomes among refugees. Maintain enhanced surveillance in high-entry transit settings to enable early detection of MDR spread.
Van Hout *et al.* (2021)^14^	Hospital, Netherlands	All patients admitted to hospital, including asylum seekers and recently sheltered migrants (*n* = 84 485)	MDR organisms	Delivery arrangement: admission screening	Low detection yield; high workload with minimal clinical benefit	Reconsider routine risk-based MDRO screening on hospital admission. Focus transmission-based precautions on known MDRO carriers (via EMR labelling). Maintain and strengthen hospital-wide MDRO surveillance systems Adapt screening strategies to local AMR prevalence and IPC infrastructure.
Urth *et al.* (2005)^15^	Community, Denmark	Danish families, including immigrant families (*n* = 46)	MRSA	Implementation strategy: home-based decolonization protocol	Successful decolonization; no onward spread to hospitals	Strengthen community-level IPC measures for MRSA. Develop clearer national policies for containment of antimicrobial-resistant organisms in the community. Reconsider and potentially broaden MRSA screening strategies beyond individuals with healthcare exposure outside Scandinavia. Enhance education and engagement of affected households to improve hygiene practices and disclosure of MRSA status during healthcare contact.
Kossow *et al.* (2018)^16^	Hospital (Germany)	Refugees admitted to hospital (*n* = 383)	MDR organisms including MRSA, MDR-GNB and VRE	Delivery arrangement: admission screening and contact isolation	No nosocomial transmission; standard hygiene deemed sufficient	Routine pre-emptive isolation of refugee patients on hospital admission may not be necessary when standard hygiene measures are applied. Policy decisions regarding isolation and screening should balance prevalence data with evidence of actual in-hospital transmission risk.

### Risk-of-bias assessment

Assessment of internal validity involved two reviewers (L.L., A.W.-W.) independently evaluating each study using tools appropriate to the study design, with any discrepancies between the primary reviewers to be resolved via discussion with a third reviewer (C.E.M.). Randomized controlled trials were to be assessed using The Cochrane Risk of Bias Tool (RoB 2).^17^ For non-randomized studies of interventions, the ROBINS-I (Risk Of Bias In Non-randomized Studies—of Interventions) was to be employed.^18^ Other non-randomized or descriptive studies would be evaluated using the appropriate Joanna Briggs Institute (JBI) Critical Appraisal Checklists.^19^ Studies were not excluded on the basis of risk of bias, as the aim of this search was to capture all available evidence.

## Results

### Study selection and characteristics

**Figure 1.**
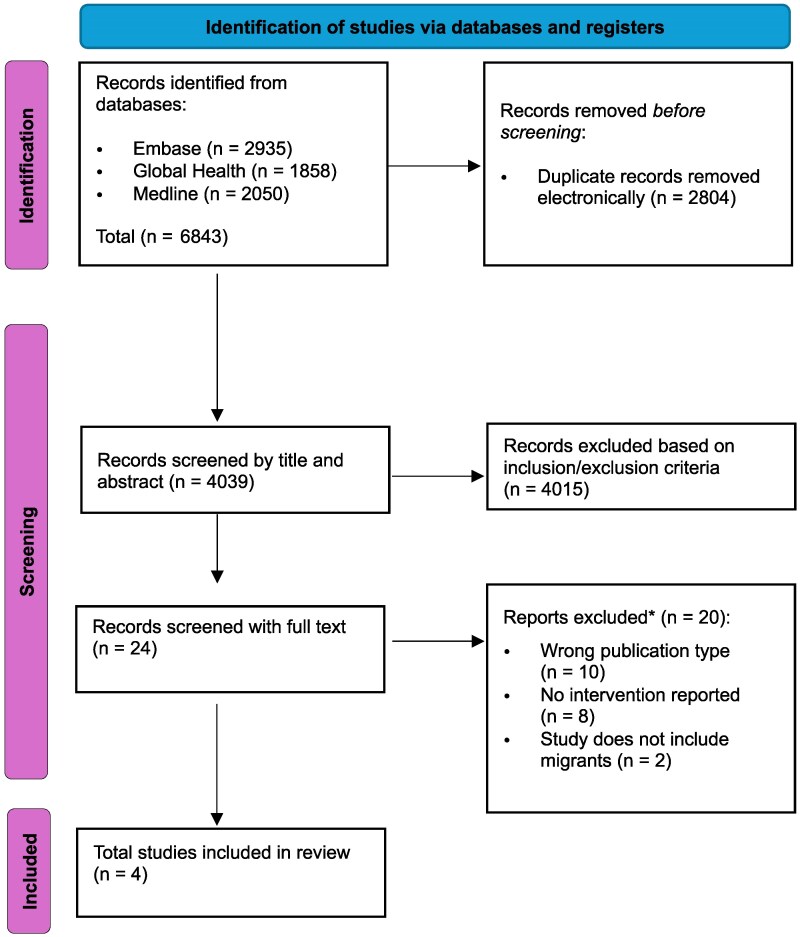
PRISMA flow chart demonstrating study selection process.

A total of only four peer-reviewed studies met the inclusion criteria following database searches in December 2025.^13–16^ The selection process is detailed in the PRISMA flow diagram (Figure 1). Primary reasons for exclusion at full-text level were ineligible publication type (e.g. review articles, dissertations), lack of interventional data or the absence of migrant-specific disaggregated outcomes.

The included studies highlight a limited evidence base on AMR interventions in migrants. Characteristics of these four studies, including setting, population demographics and reported outcomes are summarized in Table 1.

### Synthesis and quality assessment

Substantial heterogeneity in study designs, populations and outcome measures precluded meta-analysis; findings were synthesized narratively. Methodological quality was assessed using JBI Critical Appraisal tools (Table S3). Two studies (reporting prevalence and cross-sectional data) demonstrated a low risk of bias, while two case series were judged to have a moderate risk of bias.

Interventions were primarily focused on infection prevention and screening strategies. Two studies evaluated hospital-based admission screening for MDR organisms, with inconsistent clinical benefit and substantial resource implications. One study reported on syndromic surveillance in a transit setting, demonstrating feasibility but with limited evaluation of sustained outcomes. A community-based MRSA decolonization programme reported successful short-term eradication, although long-term effectiveness was not assessed.

## Discussion

### Areas of improvement for AMR interventions in migrants

#### Data collection and surveillance

A persistent limitation of interventions identified was a lack of disaggregated AMR data reported by migration-related variables including migration status, country of origin, ethnicity and housing separate to the host nation. Routine hospital and national surveillance systems rarely capture this information when reporting data, obscuring the prevalence, scale and dynamics of drug resistance in migrants. This is a documented issue, with the WHO noting that official surveillance systems often lack data on migrants.^20^ This results in a feedback loop in which inadequate resources perpetuate poor outcomes and the marginalization of migrants. This gap restricts the ability of health authorities to monitor AMR trends, evaluate intervention effectiveness and allocate resources equitably.

Few studies screened in this review specify migration type (e.g. refugee, asylum seeker, labour migrant), preventing subgroup analysis. Where available, variations in study design, intervention types and populations severely limited comparability. This has been described in recent systematic reviews, where the heterogeneity of existing studies and potential difficulties in making comparisons and drawing conclusions have been highlighted as complications in determining the AMR burden in these communities.^21^ The lack of standardized, disaggregated surveillance data remains a critical gap in global AMR monitoring, and a major obstacle to designing inclusive, evidence-based interventions.

#### Intervention design: equity and accessibility

Many of the reported AMR interventions in migrants were embedded within hospital-wide infection prevention and control (IPC) programmes, including early detection, isolation and hygiene measures.^14–16^ While these measures are crucial for hospital-acquired infections, they focus solely on the clinical setting which is inadequate to minimize AMR in these communities. Evidence suggests that prevalence of drug-resistant bacteria can be higher in community settings such as reception centres and refugee camps compared with hospitals, underscoring the need to describe and address community acquired infections in these populations.^21^

These hospital-based approaches are not designed to capture migrants’ circumstances. Refugees and asylum seekers often reside in temporary or overcrowded housing, they experience frequent relocation and barriers to healthcare such as cost, entitlement and language restrictions. These factors reduce the reach and effectiveness of strategies to minimize AMR and may contribute to further exclusion of these populations.^22,23^

The limitations in the effectiveness and reach of hospital IPC interventions highlights the missed opportunities for prevention and early diagnosis in more accessible community spaces. Community-based approaches, such as outreach or mobile clinics, may be better placed to engage migrants and implement prevention strategies. Within these settings, co-designed interventions with communities could include point-of-care diagnostics to enable timely detection and treatment. A report from a transit centre in Greece detailed the implementation of a local syndromic notification centre with active case-finding and environmental hygiene measures, allowing early detection of clusters and tailored responses to drug-resistant Shigella infections.^13^ This initiative represents one of the few examples of a community-based intervention identified in this systematic search. Such initiatives demonstrate the potential of community-level platforms to overcome structural barriers. However, such evidence remains scarce, with few rigorously evaluated migrant-specific interventions outside hospital settings reported in the literature.

#### Outcome measurement and evidence quality

This review found the current literature is limited in its reporting of outcomes in AMR-related interventions targeting migrants. Several studies describe screening, decolonization or hygiene-based interventions; outcome data, particularly for migrants, are often more limited. Two hospital-based studies reported on infection trends in migrants, with the main outcome being the absence of onward spread. Outcome reporting was generally limited in scope, often focusing on the prevention of transmission rather than longer-term health effects or individual-level effects on migrants. Similarly, studies evaluating screening approaches have provided useful insights into the feasibility and operational implications but have not assessed effectiveness or any direct benefit for migrants.^14,16^

Two community-based interventions also reported trends among migrants, with the main outcome similarly being the absence of onward transmission. One surveillance initiative documented limited person-to-person clustering followed by no further spread, while a community-level decolonization programme of a high-priority pathogen (methicillin resistant *Staphylococcus aureus*) achieved high initial clearance of carriage and prevented transmission into healthcare settings.^12,16^ However, in both cases, outcome reporting was relatively descriptive and results were presented in aggregate format, limiting the extent to which migrant-specific impacts could be discerned.

Taken together, these studies predominantly report microbiological outcomes (e.g. transmission events, strain clustering) rather than patient-centred, clinical or long-term effects. In many cases, the primary purpose was to reduce transmission risks to the wider host population, with migrant health outcomes not placed at the centre of evaluation. This reflects broader challenges in intervention design and reporting, including limited subgroup analysis and lack of equity-oriented frameworks. As a result, the current evidence base remains fragmented and provides only limited guidance for developing AMR minimization strategies that directly address the needs and health outcomes of migrants in an equitable way.

### A path forward: targeted interventions for a global challenge

In the context of increasing global migration, a rise in AMR and lack of new drugs to treat infections, there is an urgent need for sustainable, migrant-inclusive, public health strategies to strengthen responses to AMR.

Interventions identified in this study include both community- and hospital-based care. However, without a robust evidence base to inform them, policies risk failing to achieve their intended impact and cannot be meaningfully assessed against equity-oriented metrics. Specifically, future interventions require greater insights in three key areas: equitable and accessible intervention design that actively overcomes structural barriers; adequate data on AMR burden and intervention efficacy; and migrant-centred outcomes.

Dedicated migrant health services in areas of high population movement may provide one potential approach for prevention and minimization of AMR. These services should incorporate targeted antimicrobial stewardship protocols and context-sensitive IPC measures, through co-creation with all stakeholders, while accounting for migrants’ regions of origin and prevailing AMR patterns. Clinical pathways may require a lower threshold for switching antimicrobials following poor response, alongside prioritization of culture testing for high-risk groups such as migrants. Strengthening access to point-of-care diagnostics and ensuring routine microbial testing is integrated into clinical pathways at health facilities in high-migrant areas could reduce diagnostic delay, inappropriate prescribing and onward transmission. Crucially, interventions must be co-designed with migrants and delivered in ways that do not create barriers to care, including fears of legal or social repercussions.

The potential for community-based policies to provide a targeted and effective approach is strongly supported by the role of primary care systems. It could also redress health inequities for migrants.^23^ As a first point of contact, primary care is uniquely positioned to mitigate barriers to access. Furthermore, transit centres are in a position to treat high proportions of migrants and are potentially better suited for targeted interventions than hospital-wide policies. Functioning as a gateway and coordinator of care, a robust primary care system provides a good platform for implementing the targeted antimicrobial stewardship and IPC measures necessary to address the specific needs of migrants.

### Measuring impact: towards standardized and ethical evaluation

To support evidence-based policymaking, interventions must be rigorously evaluated using standardized outcome measures that include both AMR and antimicrobial use (AMU) indicators. Data collection should be embedded into routine practice within migrant healthcare settings, ensuring approaches are culturally sensitive and ethically sound.

Potential outcome measures include:

- AMU indicators:
  - Rates of appropriate antibiotic selection and prescribing, ideally aligned with national and/or local guidelines and WHO AWaRe classification principles.^24^
  - Time to initiation of appropriate antimicrobial therapy.
- Local incidence and prevalence of multidrug-resistant organisms;
- Treatment outcomes disaggregated by migrant migrant-related variables (e.g. legal status, duration of residence, healthcare access), housing conditions and region of origin.

Mapping patient pathways for the different migrants can help identify delays and gaps in care, while evaluation of feasibility and community engagement is essential to ensure interventions are effective in real-world contexts to minimize AMR. Without such insights, policies risk failing to achieve their intended impact and cannot be meaningfully assessed against equity-oriented metrics.

### Limitations

This review has several limitations to discuss. First, by focusing on interventional studies with measurable outcomes for migrants, it likely underrepresents the broader evidence base, as data disaggregated by migration status are often lacking. Small migrant cohorts within larger studies may therefore be overlooked, particularly in community-based settings where migration status is not routinely recorded. Second, the emphasis on high-quality, measurable data may bias findings towards well-resourced clinical settings, underrepresenting interventions in informal or resource-constrained regions. Third, the search was restricted to peer-reviewed studies across three major databases; exclusion of grey literature may have omitted impactful, localized interventions by non-governmental or informal organizations. Finally, the small number of eligible studies (*n* = 4) and heterogeneity in the data prevented meta-analysis and the application of a standardized risk-of-bias tool.

### Conclusion: from omission to inclusion in the AMR response

The growing threat of AMR requires responses that are both evidence-based and equity-driven. The limited inclusion of migrants in existing interventions reflects systemic gaps in public health planning rather than a lack of research interest. Addressing AMR in migrants demands high-quality, disaggregated surveillance data, which feeds into National Action Plans for AMR, and that are contextually appropriate models of care and robust evaluation of interventions. Embedding equity within AMR policy is essential not only to protect migrant health, but also to strengthen health systems and sustain global progress against AMR.

## Supplementary Material

dlag059_Supplementary_Data

## Data Availability

No additional data was generated in this study.

## Supplementary data

Tables S1–S3 are available as Supplementary data at JAC-AMR Online.
