# Supplementary material for: Interventions to address antimicrobial resistance in migrants: a systematic review
Source: JAC Antimicrob Resist. 2026 Apr 30;8(3):dlag059. doi: 10.1093/jacamr/dlag059 (PMC13132166; doi:10.1093/jacamr/dlag059)
Supplement: dlag059_Supplementary_Data [file dlag059_supplementary_data.docx]

**Supplementary Data**

Supplementary Table S1: Search strategy PICO framework

| # | Search statement |
| --- | --- |
| 1. | (alien* or “asylum seek*” or displaced or emigrant* or emigrat* or foreign* or immigra* or migrant* or newcomer* or “non-citizen*” or “non native*” or refugee* or traffick* or undocument*).ti,ab,tw. |
| 2. | (action* or aware* or benchmark* or campaign* or nsensi* or control* or educat* or goal* or guideline* or hygiene or implement* or initiative* or interpret* or interven* or legislat* or method* or outreach or policies or policy or practice* or prevent* or procedure* or program* or promot* or protocol* or regulat* or screen* or service* or steward* or strateg* or train* or translat* or vaccin* or WASH).ti,ab,tw. |
| 3. | ((antimicrobial* or antibiotic* or drug* or microbial* or pathogen*) adj3 (resistan* or “non susceptib*” or “decreased susceptibility” or “decreased sensitivity*” or insusceptib* or insensitive*)).ti,ab,tw. |
| 4. | (beta-lactamase* or ESBL or CTX-M or AmpC or MBL or “methicillin-resistant Staphylococcus aureus” or MRSA or AMR or XDR or MDR or ARB).ti,ab,tw. |
| 5. | (1 and 2 and (3 or 4) |
| 6. | Limit 8 to yr= “2000 – Current” |

Supplementary Table S2: Inclusion and exclusion criteria

| Criteria | Inclusion | Exclusion |
| --- | --- | --- |
| Study design | Empirical studies reporting an implemented intervention (programme, policy, or practice) intended to influence AMR prevention, diagnosis, prescribing, stewardship, or treatment (EPOC-defined). Randomised, non-randomised, and mixed-method designs eligible. | Descriptive or surveillance-only studies without an implemented intervention; prevalence studies; modelling studies without implementation; laboratory-only studies; editorials, commentaries, and protocols. |
| Population | International migrants (UN definition: living outside country of birth), including short-term (3–12 months) and long-term (>12 months). Migrant status explicitly stated or identifiable by country of birth. Mixed populations included only if migrant outcomes disaggregated. | Internal migrants. Mixed populations without disaggregated migrant data. Unclear migrant status or non-migrant populations only |
| Pathogen focus | WHO Critical or High Priority AMR pathogens. | Tuberculosis. Non-AMR pathogens.or non-WHO Critical or High Priority AMR pathogens. |
| Intervention | Any implemented measure intended to influence AMR prevention, screening, infection control, antimicrobial use, or treatment. | No active intervention; laboratory-only or diagnostic accuracy studies without intervention. |
| Outcome | Measurable AMR-related outcomes, including antimicrobial use (AMU) indicators, resistance rates, clearance of carriage, infection incidence, prescribing appropriateness, treatment outcomes, or transmission reduction. | No measurable clinical or AMR-related outcomes. |

Supplementary Table S3: Risk of Bias Assessment using JBI Critical Appraisal Tool

| Paper | Critical Appraisal Tool | Score |
| --- | --- | --- |
| Georgakopoulou et al | JBI Critical Appraisal Checklist for Case Series | Moderate risk of bias (7/10) |
| Urth et al | JBI Critical Appraisal Checklist for Case Series | Moderate risk of bias (7.5/10) |
| Van Hout et al | JBI Critical Appraisal Checklist for Analytical Cross Sectional Studies | Low risk of bias (6/8) |
| Kossow et al | JBI Critical Appraisal Checklist for Studies Reporting Prevalence Data | Low risk of bias (10/10) |
